# Health beliefs of school-age rural children in podoconiosis-affected families: A qualitative study in Southern Ethiopia

**DOI:** 10.1371/journal.pntd.0005564

**Published:** 2017-05-25

**Authors:** Abebayehu Tora, Getnet Tadele, Abraham Aseffa, Colleen M. McBride, Gail Davey

**Affiliations:** 1 Department of Sociology, College of Social Sciences, Addis Ababa University, Addis Ababa, Ethiopia; 2 Department of Sociology, College of Social Sciences, Wolaita Sodo University, Sodo, Ethiopia; 3 Research and Innovation Division, Armauer Hansen Research Institute/ALERT, Addis Ababa, Ethiopia; 4 Department of Behavioral Sciences and Health Education, Rollins School of Public Health, Emory University, NE, GCR 564, Atlanta, Georgia; 5 Wellcome Trust Centre for Global Health research, Brighton and Sussex Medical School, Falmer, Brighton, United Kingdom; Michigan State University, UNITED STATES

## Abstract

**Background:**

Several studies have suggested investigation of health beliefs in children to be an important pre-condition for primary prevention of disease. However, little effort has been made to understand these in the context of podoconiosis. This study therefore aimed to explore the health beliefs of school-age rural children in podoconiosis-affected families.

**Methodology/Principal findings:**

A cross sectional qualitative study was conducted in March 2016 in Wolaita Zone, Southern Ethiopia. Data were collected through in-depth individual interviews (IDIs) and focus group discussions (FGDs), with a total of one hundred seventeen 9 to15-year-old children recruited from podoconiosis affected families. The study revealed various misconceptions regarding risk factors for podoconiosis. Most children believed barefoot exposure to dew, worms, snake bite, frog urine, other forms of poison, and contact with affected people to be major causes of the disease. Their knowledge about the role of heredity and that of long term barefoot exposure to irritant mineral particles was also weak. Though most participants correctly appraised their susceptibility to podoconiosis in relation to regular use of footwear and foot hygiene, others based their risk perceptions on factors they think beyond their control. They described several barriers to preventive behaviour, including uncomfortable footwear, shortage and poor adaptability of footwear for farm activities and sports, and shortage of soap for washing. Children also perceived low self-efficacy to practice preventive behaviour in spite of the barriers.

**Conclusion/Significance:**

Health education interventions may enhance school-age children’s health literacy and be translated to preventive action. Overcoming practical challenges such as shortage of footwear and other hygiene facilities requires other forms of interventions such as livelihood strengthening activities. Linking podoconiosis-affected families with local governmental or non-governmental organizations providing socio-economic support for households may assist school-age children in those families to sustainably engage in preventive behaviours.

## Author summary

Podoconiosis is an example of a lifestyle-related disease that develops after childhood and affects millions of people with little experience of preventive behaviour. It is caused by prolonged barefoot exposure to irritant mineral particles, accompanied by inherited susceptibility. Children with a family history of podoconiosis are highly vulnerable to the disease. Several studies have underscored the importance of studying health beliefs in children for the early control of diseases that arise from risky behaviour and habits established in childhood, which continue into adulthood. This study attempted to explore the health beliefs of school-age children in podoconiosis affected families. The forms of health beliefs addressed in the present study were knowledge of podoconiosis risk factors and perceptions of severity of and susceptibility to the disease, benefits of and barriers to engaging in preventive actions such as footwear use and feet hygiene, and self-efficacy to perform preventive actions regardless of perceived barriers. Various forms of misconceptions and inaccurate risk perceptions were recorded. Participant children also reported several barriers that limited their engagement in preventive actions and low confidence to overcome them. These findings imply that school-age children at high risk of podoconiosis might benefit from an intervention that improves their knowledge about podoconiosis and enhances their self-efficacy to sustainably perform preventive behaviours.

## Introduction

Podoconiosis is an example of a lifestyle-related disease that develops later in life and affects millions of people with little experience of preventive behaviour. It is non-infectious (and thus also termed ‘non-filarial elephantiasis’) and is characterized by bilateral swelling of the lower legs, commonly affecting people in the economically productive age groups [1,2]. In Ethiopia, over 1.5 million people are believed to live with podoconiosis [3]. Evidence to date indicates that the combination of inherited genetic susceptibility and barefoot exposure to soil rich in irritant mineral particles contributes to the cause of podoconiosis [2,4]. An estimate of heritability of podoconiosis is 63% while the risk ratio of siblings in affected families is 5 times higher than their counterparts in the general population [2]. Luckily, genetically susceptible individuals can entirely prevent the disease if they consistently protect their feet from exposure to irritant particles by wearing shoes starting at young age [5]. However, few children in podoconiosis-affected families engage in preventive behaviours such as regular use of footwear and foot hygiene in spite of their higher susceptibility to the disease. In the most recent study in an endemic setting in Ethiopia, the proportion of preschool children reported to have “all day, every day” use of footwear was only 31% [6]. Another study also reported poor hygiene among children [7].

Previous studies among adults in communities endemic for podoconiosis have reported higher level of misconceptions regarding the cause and prevention of podoconiosis [6–11], and discussed the implications of the misconceptions to disease prevention behaviour and interpersonal interactions [6]. The beliefs that podoconiosis is contagious, caused by worms in the soil, indiscriminately inherited among relatives, caused by evil eye, curse, witch, or cold weather [8,9] were found to have negative consequences on preventive behavioural choices and interpersonal interactions [10]. The perceptions of adults regarding their own and children’s susceptibility to the disease were also reported to be inaccurate [6,11]. The perceptions that footwear does not permit farm activities and other duties, is uncomfortable for walking in the mud, smells bad in the hot season, wears out too quickly, softens the feet, and should be preserved for special events have all been identified as factors discouraging optimum use of footwear among people at high risk for the disease [11,12]. However, most of these studies focused only on adults. The studies that have investigated preventive behaviour among children [6,7,13] have explained it based on the parents’ health beliefs. Children are perceived as “active, purposeful beings who make sense of their world and contribute substantially to their own development” [14], and whose cognitive developments occur intensively within the age of 7–15 years [15–17]. Researchers have acknowledged increasing levels of social autonomy of school-age children as they spend more time away from home with less parental supervision. This gives them the chance to develop independent beliefs about health [18].

Several studies have underscored the importance of investigating the dimensions of health beliefs in school-age children, particularly for control and prevention of diseases that arise from behaviour and habits established in childhood and continue to adult life [18, 19–23]. The formation of values and behaviour in early childhood necessitates understanding of health beliefs of children [19]. This is supported by other studies which argue for the establishment of accurate beliefs about health in early childhood as habits in childhood are predictive of habits in adulthood [20–22]. Investigating health beliefs in children is also thought to enable better understanding of the impact of health education on the modification of health beliefs and encouragement of preventive behaviour [18,19]. Knowledge of the health beliefs of school-age children can be used to engage them as health messengers to their families and peers. A growing body of thought supports the belief that school-age children are not just passive recipients of health information. Rather, they can act as change agents who positively influence the behaviour of others in their communities through communicating health messages [24, 25]. Investigating the health beliefs of school-age children may not only help promotion of footwear use for preventing podoconiosis, but also prevention of other neglected tropical diseases contracted through the feet. To our knowledge, no studies have actively involved children of this age group in the study of their health beliefs in the context of podoconiosis. The main purpose of the present study is therefore to explore various forms of health beliefs in school-age children.

## Methods

### Ethical consideration

Ethical approval was obtained from the ethics committee of the Armauer Hansen Research Institute (AHRI) (Project reg. No. P035/15) and College of Health Sciences, Addis Ababa University (Protocol number 047/15/Ext). The Wolaita Zone Administrative Bureau gave written permission to work in the community. The Mossy Foot International also allowed their outreach clinic site staff to help in the identification of study participants. Individual participation of children and group interviews was guided by the international guidelines for involving children in research [26–28]. A developmental psychologist helped during the recruitment and interviews to make sure that the content and format of questions was appropriate to the age and cognitive level of the child. Informed consent was obtained from caregivers and children also gave their assent to participate in the study. Taking into account likely difficulties understanding written consent forms due to low literacy [29], a conversational style oral presentation of consent information was made in the local language to caregivers and children. Caregivers confirmed their permission for a child to participate in the study by signing or thumb-printing on the consent forms. Children expressed their assent verbally in the presence of their caregivers as a witness, to ensure the assent process is without any coercion. The use of verbal assent to children was approved by the ethics committee.

### Study setting

**Fig 1:**
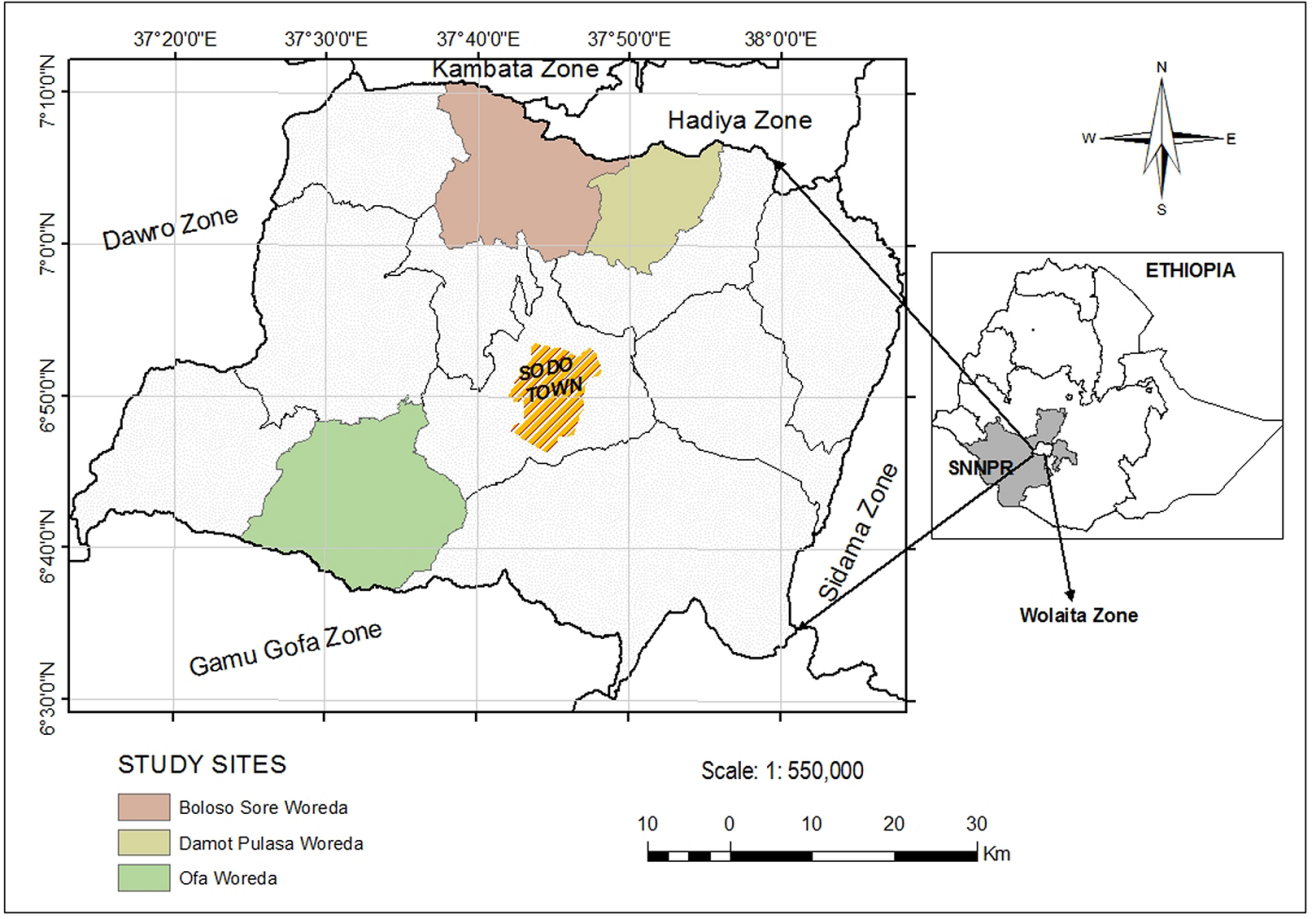
Map of study area.

The study was conducted in Wolaita Zone, one of the thirteen zones in Southern Nation Nationalities and Peoples Regional State (SNNPRS). Wolaita Zone is located in the south-west of Ethiopia roughly between 6.30–7.10 N and 37.10–38.10 E, latitude and longitude respectively (Fig 1). According to the 2007 census report, the total population of the area was around 1.7 million; of whom 83.2% resided in rural areas. The dominant means of living is subsistence agriculture [30]. In this Zone, the point prevalence of podoconiosis has been calculated at 5.46% [31]. Since 1998, Mossy Foot International (MFI) (formerly, the Mossy Foot Treatment and Prevention Association), an international non-governmental organization, has been offering community-based prevention and control activities against podoconiosis in 15 outreach sites located at 15 to 65 km from the head office in Wolaita Sodo. Clinics in all outreach sites are run by community podoconiosis agents (CPAs) who are themselves patients, and social workers recruited from the local community. The MFI reaches the wider community through a group of network members who provide voluntary services of awareness and demand creation in collaboration with site workers. The organization has been serving over 30,000 registered patients for over a decade [32].

### Study design and sampling

A cross sectional qualitative study was conducted in March, 2016 using in-depth individual interviews (IDIs) and focus group discussion (FGDs) methods. A purposive sampling technique was used to select three study sites with large numbers of registered patients and a relatively long history of establishment. The selected sites were Damot Pulasa Woreda (district), Boloso Sore Woreda and Ofa Woreda. Study site staff members helped identify affected families and children eligible for interviews in those families. A theoretical sampling technique was used to determine the number of participants in the study, i.e. the process of sampling that continues until theoretical saturation is reached [33]. The major inclusion criteria for children were being part of a podoconiosis-affected family and age 9–15 years. Podoconiosis-affected families having at least one child between the age of 9–15 years were identified through the MFI site workers. One child per household was selected either for IDI or for FGD. None of the selected child participated in both activities. Children affected by podoconiosis or other forms of physical impairment were excluded from the study. Focus group discussions were disaggregated by gender: three with boys and three with girls. Twelve children participated in each FGD, giving a total of 72 participants. A total of 45 IDIs were held with children: 15 in each of the three sites. Together, 117 children participated in individual and group interviews.

### Data collection

Semi-structured questions were developed in English and translated into the local language, *Wolaitatto* (AT is the native speaker). Children were interviewed individually during home visits while FGDs were held in a place proximate for children coming from surrounding villages. All interviews were digitally recorded. Before assessing the beliefs of children about podoconiosis in both IDIs and FGDs, the mental image of children regarding their recognition of podoconiosis as a disease and its manifestations was assessed through drawings and their verbal description of the pictures they drew. Drawing exercises have been suggested as a tool to understand children’s imagery of disease and to assess their knowledge and conceptions [34,35]. To prompt recalling, the interviewer used the local term “na’u gediya kitisiya hargiya”, literally “disease that causes bilateral swelling of feet”. This term was used instead of common local terms such as “Kita”, “Inchricha” [8], which were reported as derogatory and entailing demeaning connotation against affected persons [29]. Participant children were asked about their thoughts regarding the cause of podoconiosis, their perceptions of severity of disease, their appraisal of susceptibility to the disease, the advantages and disadvantages of various types of footwear, their perceptions of barriers to regular use of footwear and their self-efficacy beliefs to use footwear regularly in spite of these barriers. In the middle or at the end of FGDs, role plays were performed by children, which boosted their confidence to talk openly what goes on within the family and in the community. Whenever children found a given question difficult to understand, guidance and repeated clarifications were used to facilitate responses. While individual interviews lasted a maximum of 30 minutes, it took 1 hour to complete each FGD. At the end of the interviews, participants were provided brief information about the causes of podoconiosis and the role of consistent use of footwear in preventing the disease. After every interview, children received a piece of soap, a pen and a note book as compensation for their time, and this was suggested in a previous study as ethically appropriate if made in consultation with community members in the study setting [29].

### Data analysis

Data were transcribed and translated into English and imported to NVivo software version 11 for analysis. Both deductive and inductive approaches were used to analyse the data. Deductive coding of themes in the data was based on Health Belief model constructs such as knowledge, perceived severity, perceived susceptibility, perceived benefits and barriers, and perceived self-efficacy. The Health Belief Model (HBM) provides a useful framework to understand and explain health beliefs in association with disease preventive behaviour [36–40]. A number of studies have used the health belief model to explain health beliefs of young and adolescent children [18,19,41,42].A grounded theory approach was used to inductively identify newly emergent themes and subthemes as coding process proceeded. Grounded theory refers to a set of integrated and inductively generated concepts, categories, and themes that are formulated into a logical, systematic and explanatory scheme [33].

## Results

### Knowledge about podoconiosis

#### Knowledge of symptoms of podoconiosis

Participant children were asked to draw a picture of feet affected by podoconiosis if they had a clear mental image about the disease and its symptoms without being assisted by further descriptions of the disease. Except few who were shy to draw pictures, most of the participants pictorially demonstrated the physical manifestations of the disease. To further verify the children’s conception that podoconiosis was distinct from similar attributes of other diseases, they were further asked to verbally describe its manifestations and mention someone with similar symptoms of swelling in their family. Except the participants whose family member had already recovered or at the earliest stage of the disease, most children suggested their family members affected by the disease. They also indicated attachment of affected person they mentioned with the local organization receiving treatment and the type of care he or she does to his or her feet.

Yes, I know. My aunt has feet swelling. And, my grandmother is affected too. My aunt receives treatment from the organization [referring to MFI] every month for herself and her mother. They live with us in the same house (IDI participant, girl, 13 years, grade 6).

They were also able to explain how the swelling happened.

It swells up. And sometimes, the sides of feet burst and excrete fluids. It causes illness in that way. It affects two legs. Both legs swell and cause illness. (IDI participant, boy, 11 years, grade 5)

#### Knowledge of risk factors and preventive measures

The knowledge of children about risk factors of podoconiosis was discussed from three major domains: knowledge about behavioral and environmental risk factors, knowledge about heredity as a risk factor and knowledge about preventive measures.

#### Knowledge about behavioral and environmental risk factors

Children raised a number of behavioral and environmental risk factors as causes of podoconiosis such as barefoot exposure to “worms”, “germs”, “poison”, “dew”, “chilly weather”, “snake bite”, “frog urination”; injuries by sharp things particularly “rusty metal, blade, needle, and spine”; walking barefoot on “pond water”, “jarring road”; exposure to “mich” [a local name for illness related to heat], “insulting an affected person” and “lack of exposure to sun in childhood”.

The major reason for the cause of swelling is that when we walk barefoot in the field, our feet may be injured by sharp things. When we come home, we may use rusted blade or needle to take out the spine. Because of that our feet may be poisoned and become swollen (FGD participant, boy, 14 years, grade 7).

Walking on morning dew without shoes, and exposure to snake, worm, and frog bite can cause feet swelling (FGD participant, girl, 12 years, grade 3)

Though the term “soil” or “dust” was frequently mentioned, children referred it to poison and other harmful things in the soil. None of them raised mineral particles in the soil known for causing the disease.

Because of walking barefoot, the feet trap dust through sweating, and foot swelling develops consequently. (FGD participant, boy, 12 years, grade 6)

Because of walking barefoot, worms in the soil enter into the feet and cause swelling. (FGD participant, girl, 12 years, grade 1)

The belief that podoconiosis is contagious was common among children. Most believed that podoconiosis could be transmitted by stepping where an affected person had stepped on, or by sharing shoes or needles with affected people.

When someone steps on a place where an affected person stepped, he gets the disease. (IDI participant, boy, 11 years, grade 5)

I believe that sharing shoes with an affected person can pass the disease. (FGD participant, girl, 14 years, grade 3)

#### Knowledge about heredity as a risk factor

Children rarely mentioned heredity when they were asked general questions about the cause of podoconiosis. Their immediate answers to such a question centered on non-biological risk factors such as contagion, lack of foot hygiene and barefoot exposure. The issue of heritability was raised using more probing questions. For example, “in your community, people commonly believe that podoconiosis is hereditary, what is your opinion about such a belief?” The local language used for the term ‘hereditary’ was “zariyappe latettees”. Though most participants could comprehend the concept, some participants found it difficult to understand which was expressed by either being silent or asking for clarification. Hence, the children’s understanding about the role of heredity was assessed mainly through indirect questions like “have you observed families in which two or more members are affected by podoconiosis? If so, why do you think this happens?” To further explore the children’s understanding of joint contribution of heredity and environment, they were asked: “in the same community, some people are affected by podoconiosis while others are not. Why do you think this happens?”

A range of views on heritability were held by the children, some acknowledging heritability while also stressing on preventability and others confusing heredity with infection.

One FGD participant indicated his understanding of heritability as follows:

A woman in our neighborhood said “this disease is inherited from relatives who had similar disease before”. I heard this. She said, if there is ‘ti’e’ [a local word for tumor] in the blood line, then it causes others in that line to get feet swelling”. I also think it can be inherited in this way. (FGD participant, boy, 10 years, grade 2)

Another participant also expressed his agreement with the community perception of heredity, indicating the controllability of the disease through preventive actions such as foot hygiene and using shoes.

I was shocked when I heard people saying the disease is hereditary. Since I suspect that I can be a victim in this way, I do whatever I can to protect my feet from the disease. (FGD participant, boy, 12 years, grade 4).

Others thought heritability to be one of several causes, stating chance or exposure to harmful things in the environment.

On some individuals it passes through inheritance. But for others, it appears from nowhere. There are families where two are affected. In this case, it can be said [it is] hereditary (IDI participant, boy, 12 years, grade 2).

The disease may or may not be inherited. Instead of heredity, it is stepping on harmful and rubbish things that cause the disease. (FGD participant, boy, 14 years, grade 9)

Some thought that podoconiosis can pass from affected family members to unaffected family members through contact. A girl whose grandmother, uncle, and two aunts were affected by podoconiosis was asked why she thought three people in her family were all affected by podoconiosis. She said, “….it can be inherited through [the] blood line”. However, when she was asked how her relatives could develop foot swelling, she replied, “I think it happened because they shared each other’s shoes”.

This confusion was echoed by another FGD participant -

People think the disease is hereditary when they observe that a daughter is affected like her mother. Her daughter could be affected because of sharing her mother’s shoes, and her grandchildren may get the disease in similar way. Then, people say the disease is hereditary. (FGD participant, girl, 11 years, grade 5)

The children were asked why in the same community, some people were affected and others not, to investigate whether they associated this difference to heredity. None of the participants thought heredity had a role in this, but differences within the community arose from “lack of foot hygiene and footwear use”.

#### Knowledge about preventive measures

Children commonly perceive that podoconiosis can be prevented by regular use of footwear and foot hygiene. They literally indicated that their affected family members could have prevented the disease had they worn shoes and kept foot hygiene. A 12 year old boy states,

My mother’s feet could swell because she was walking without shoes on soil and going to bed without washing feet at night, and wakes up in the early morning, and goes to work without shoes (FGD participant, grade 4).

However, most of them also endorsed avoiding contact with affected person to prevent the disease: “by not wearing their shoes”, “by not stepping where they stepped”, and “by not using the water used by the affected person while washing feet”.

#### Sources of information about podoconiosis

The major sources of information for children regarding podoconiosis were reported to parents and observation of affected persons in the family and neighborhood. None of participant children indicated learning about podoconiosis at school. The following statements indicate the sources of information for children.

I heard my grandmother saying that stepping where affected person stepped could cause the disease. (FGD participant, girl, 13 years, grade 5)

My mother said to me that wearing the shoes of affected person causes the disease. She also said that the disease could be caused by stepping on worms, and going to school without washing feet. (IDI participant, girl, 11 years, grade 3)

### Perceived susceptibility to podoconiosis

Children were asked if they thought they might or might not be at risk. Some provided logical reasons for considering themselves at risk considering exposure to risk factors in the environment and lack of preventive actions taken to reduce their exposure to these risk factors. They believed that they would be at risk if they went barefoot and were aware that their exposure could be reversed through preventive actions. If I walk barefoot and simply step on harmful things, I think my feet may also swell like that of my father. As a result, whatever they complain about poverty, I urge them to buy shoes for me. (FGD participant, boy, 13 years, grade 4)

In times when I walk barefoot due to lack of shoes or until my mother buys other shoes for me, I may get the disease. That is why I am worried. (FGD participant, girl, 11 years, grade 2)

On the other hand, misperceptions around personal susceptibility to podoconiosis were also common. Some children thought that they were at risk based on exposure to factors that are not recognized to cause podoconiosis such as exposure to snake bite and sharing of contaminated water.

I am worried because I see my father suffering from the illness related to his feet. Because of that, I don’t wash my feet with the water he used to wash his feet. (FGD participant, boy, 12 years, grade 4)

Yes I am worried because I may be bitten by a snake. Affected people say that their feet were swollen because of snake bite. (FGD participant, boy, 9 years, grade 2)

Some children never thought about the risk of getting podoconiosis. This is mainly because they related occurrence of disease to ‘God’s plan’.

No, I never thought about it. It happens only because of God’s plan. People become sick only if God permits it to happen. (IDI participant, girl, 14 years, grade 3)

Some children thought that they were not at risk and not worried of getting the disease because their feet were healthy at the time of interview. When a boy was asked whether he had ever thought about getting podoconiosis, he replied “no, I have never thought about getting foot swelling as my feet are healthy now. (IDI participant, boy, 12 years, grade 2)

### Perceived severity of podoconiosis

Participants in all individual and group interviews were aware of the negative consequences of the disease on affected individuals and their families. They noted that podoconiosis “causes illness and results in unexpected medical expenses”, “limits capacity to work”, “embarrassing”, “exposes family of affected person to starvation”, “causes illness and makes them bedridden due to swelling in groins”, “limits their ability to walk or run fast”, and “dissociates them from other people because of the bad smell and flies collected on wounds of their feet”. More importantly, they also revealed the impact of their parents’ condition on their own life, citing stress and starvation because of their parents’ illness.

When my father gets sick, I feel stressed as I observe him depressed. There is shortage of food in our house because my father cannot work like others due to his foot condition. We don’t have things others have. We don’t dress like other’s children. Our needs are not fulfilled. (FGD participant, boy, 12 years grade 4)

### Perceived benefits of engaging in preventive behavior

The participants held a positive outlook towards using footwear. They stated that using footwear protects the feet from dust, snakes, poison, dew, chilly weather, injuries by sharp things.

They protect feet from sharp objects such as spines, needles. They protect feet from cold weather and dew. (IDI participant, boy, 11 years, grade 2)

Children also clearly stated the importance of foot hygiene in preventing podoconiosis. As a FGD participant states, “if people wash feet regularly, before and after wearing shoes, they cannot get the disease” (FGD participant, boy, 12 years, grade 4).

### Perceived barriers to engage in preventative behavior

The perceived discomfort of footwear, particularly in hot weather, was commonly thought to be a barrier to regular use. Children associated closed rather than open shoes with discomfort in hot weather. They repeatedly stated that using closed footwear in hot weather caused nail dystrophy and ‘mich’.

We need to wear sandal shoes in hot (day) time to keep feet fresh. If we wear closed shoes in hot times, they pull out our toenails. (FGD participant, boy, 13 years, grade 7)

Closed shoes can expose feet to “mich” if it is taken off in hot time. As a result feet can swell up. (FGD participant, boy, 11 years, grade 4)

On the other hand, girls tended to perceive the disadvantages of both open and closed footwear. They thought that the regular use of sandals caused heel fissures while the regular use of closed plastic boots damaged the toenails. They recommended changing between different types of footwear for better foot health.

If we don’t use both interchangeably, open shoes cause heel fissures. *Bajaj* [local name for closed plastic boots] causes nail dystrophy in hot time. Hence, we wear sandals when we go to distant places and during hot times. [FGD participant, girl, 13 years, grade 5]

Most children also said that they felt uncomfortable using any type of footwear when they engaged in farming activities.

I don’t feel comfortable to work in the garden with shoes. I only wear shoes when I go to the forest to collect fuel wood to protect my feet from spines. (FGD participant, boy, 10, grade 2)

Children also said it was difficult to do sport wearing shoes. While some were worried about shortening of the shoes’ life, others were more concerned about injuring themselves.

I feel comfortable to play barefoot. When running with shoes, it exposes to slipping. (FGD participant, girl, 13 years, grade 5)

Another problem was the limited number of pairs of footwear they owned. Those who had a single pair of shoes kept them for special occasions such as school or church. Some children were concerned about the economic situation of their parents and refrained from wearing shoes regularly so as to preserve the ones they had. The poor quality of shoes purchased for children was also reported to negatively influence the motivation of children to use them regularly.

I have only this pair [open plastic]. I wear them when I go to school and church. I walk barefoot after I come home from those places to preserve them. (IDI participant, girl, 12 years, grade 2)

My father doesn’t buy the type of shoes I want. I ask him to buy “shera taketa” [canvas football boots]. But he buys only “Kito”. When I complain about it, he says “you can leave it if you are not pleased to wear”. (FGD participant, boy, 10 years, grade 2)

Regarding foot washing, most children indicated that they washed their feet before putting on shoes, before going to bed in the evening and after performing certain activities barefoot. Almost all of the children reported that they washed their feet at least once a day. They did not perceive barriers to washing their feet regularly, except discipline or consistency. The availability of water nearby and the encouragement of parents were reported to be enabling factors. However, when observed, the participant children’s feet were not as clean as expected since they rarely used soap.

There is shortage of soap. A small piece of soap costs one birr or two birr. Most of the time, I wash my feet without soap. (FGD participant, girl, 13 years, grade 5)

I usually wash my feet without soap. My parents buy soap for clothes, not for feet. There is also no trend of washing feet using soap. When I get soap, I wash my hair with it. I never use soap for my feet. (IDI, boy, 12 years, grade 2)

### Perceived self-efficacy to use protective footwear regularly

As discussed earlier, because of the discomfort of wearing closed shoes for farm activities and hot weather, children tended to instead wear either open (less protective) types of footwear or to walk barefoot. They were asked if they thought they could use closed footwear for all conditions regardless of the perceived challenges. Most said they could not wear closed shoes while farming because they were too heavy and cause bad smell.

I cannot wear shoes in the farm field. It is not comfortable to work with shoes because soil enters into it and makes it heavy. (IDI participant, boy, 11 years, grade 5)

I don’t think I can wear closed shoes [referring to closed plastic boots he wore] for the whole day. It burns. And it will expose to ‘mich’ and can become a reason for swelling of feet. (FGD participant, boy, 10 years, grade 2)

On the other hand, for some participants, self-efficacy depended on the type of closed footwear. For instance, most children thought that they could use canvas or ‘sieve’ plastic boots (with ventilation holes) in all seasons or times of day. They thought that these boots let air circulate around the feet in hot weather and are comfortable for cold weather since they cover the whole foot. Canvas shoes were also thought to be good under all conditions.

Yes, I can wear them (referring to sieve plastic boots) in hot weather because my feet can get fresh air through the small holes. I can use it throughout the day. (FGD participant, boy, 14 years, grade 6)

I cannot use this ‘taketa’ [plastic football boots] in hot time because it causes bad smell. But, I can wear ‘shera chama’ [canvass shoes] for the whole day. (FGD participant, boy, 12 years, grade 5)

## Discussion

Using the Health Belief Model, this study attempted to explore children’s knowledge regarding the cause and prevention of podoconiosis, their perceptions about susceptibility to and severity of the disease, the benefits of and barriers to engaging in preventive behaviour and their self-efficacy to perform preventive behaviour in the face of perceived challenges.

Participant children were found to have intimate knowledge of podoconiosis symptoms, which they expressed best in self-drawn pictures and verbal descriptions. The use of a simple term in the local language that is equivalent to the scientific name of the disease also helped children distinguish the attributes of the disease from other related diseases. However, some younger children, and children with fully-recovered family members or those at the early stages of the disease struggled to name podoconiosis in the local language and consequently faced difficulties drawing pictures. There were also some participants who were too shy to draw pictures and thought they lacked the skill to do so. This implies that using illustrative pictures or photos of the disease in addition to local language is necessary before proceeding to other activities such as interviews with children.

Various understandings regarding the cause and prevention of podoconiosis were apparent in interviews. The most important domain in which children’s knowledge was assessed was their understanding of behavioral and environmental risk factors. Children mentioned exposure to several environmental factors such as snakes, frogs, worms, germs, cold weather, injuries by sharp objects and rusty metals, etc. as causes of the disease. Other kinds of behaviour such as sharing shoes and household equipment, insulting affected people or jumping over a podoconiosis-affected leg were also mentioned as causes of the disease. Barefoot exposure to soil was also raised by children as a cause of podoconiosis though the role of mineral particles in the soil was mentioned infrequently. This is congruent with previous studies which reported poor knowledge of adult community members about mineral particles in the soil as a causal agent of podoconiosis [8–10,12,43,44]. Regarding preventive measures, children were well aware of the importance of footwear and foot hygiene. A previous study in Wolaita also reported large numbers of community members believing that podoconiosis was preventable through good personal hygiene and wearing shoes [8]. However, as children also endorsed contagion and other behaviour as causes, they may not intend to use footwear and keep feet hygiene and prioritize these over other actions such as avoiding contact with affected persons.

The other important domain in which children’s knowledge of podoconiosis cause and prevention were assessed was their beliefs about heredity. Unlike adults in the same study setting who immediately mentioned heredity in relation to podoconiosis in interviews [8,10], children under the age of 15 were less likely to have ideas about the issue of heredity in their short memory. It was only through further probing questions that children were able to discuss heredity in the present study. The use of questions about family clustering of the disease and its disproportionate prevalence in the community helped identify the children’s intuitive understanding of heredity in association with podoconiosis. These questions revealed misinterpretations of heredity among children. Though some children believed that heritability could be mitigated through regular use of footwear and foot hygiene, they had no clear conception of what actually was inherited, mentioning attributes like ‘tumor in the blood line’. Others confused heredity with contagion. Similar misconceptions about heredity have been reported in a previous study [10].

Studies have underlined the importance of accurate understanding of heredity and its link with mineral particles in the soil for self-motivated and self-regulated preventive actions among people at high risk for podoconiosis [10–13]. Inaccurate understandings of the concept of heredity and its role in the cause of the disease contribute to the perceived inevitability of podoconiosis, which is reflected in endorsement of risky behaviors [10]. The other form of inaccurate understanding of the role of heredity is an absolute denial of its role, which is reflected in the endorsement of environmental determinism [10,44]. However, a health education intervention with culturally and linguistically appropriate genetics information for adults suggests the possibility of enhancing genetic literacy in low income rural settings [44]. School age children may also benefit from similar health education interventions tailored to their cognitive scope.

In the study setting, there are limited circumstances in which children get information about podoconiosis in schools. They construct knowledge of podoconiosis through communication with their parents or observation of illness experiences of affected family members. As most adults in podoconiosis-endemic communities were reported to hold high levels of misconceptions about the disease [10], children may learn these misconceptions from their parents. If not addressed, this may contribute to the intergenerational perpetuation of misconceptions about the disease in communities highly endemic for the disease.

The misconceptions children held about the risk factors of podoconiosis were also reflected in their appraisal of their susceptibility to the disease. Some children believed that they were not at risk, because their feet were healthy at the time of interview, which shows lack of understanding of the onset of the disease. Others associated the risk of getting podoconiosis with bad luck or predisposition, which may also limit their motivation to engage in or maintain preventive action. Such a perception was also reported as common among adults in podoconiosis-endemic communities and acted as a barrier to preventive actions against podoconiosis [11,12]. As children associate their perception of susceptibility to podoconiosis with exposure to poison in the soil, and contact with and insults against affected people, they may consider their exposure to these risk factors beyond their control. This may limit their commitment to engage sustainably in preventive behaviours. On the other hand, though not realistic in the case of podoconiosis, the children’s worries related to exposure to other harmful things such as snakes in the environment may be used in framing health messages. This may motivate them to use protective footwear not only to prevent podoconiosis, but also several barefoot related neglected tropical diseases affecting children of the school age.

Children perceived various benefits of using footwear and practicing foot hygiene, including prevention of podoconiosis. However, they perceived several barriers including that footwear was uncomfortable, particularly under circumstances like hot weather, farming and sports activities. Similar barriers have also been reported in previous studies on adults [11,12]. Children considered closed shoes to be cumbersome, potentially smelly and likely to cause nail dystrophy. They related problems concerning footwear not only with the number of pairs of footwear they owned, but also the adaptability and acceptability of footwear they own. Children with multiple pairs of footwear tended to report stronger self-efficacy beliefs to use footwear in all conditions of daily life. Financial constraints were reported to be the major obstacles limiting parents’ capacity to provide acceptable and adaptable shoes [12]. The effect of poverty was also reflected in the children’s use of soap for washing their feet, forcing them to prioritize soap for washing the face and clothes. Hence, these practical challenges should be considered together with their misconceptions about the disease during health promotion activities.

### Strengths, limitations and future directions

To our knowledge, this study is the first to address health beliefs of school-age rural children in podoconiosis-affected families. We have explored dimensions of health beliefs that are important to public health intervention in relation to this condition and other Neglected Tropical Diseases. However, the findings of this study may not be generalizable to other settings as the participants belong to a homogenous cultural background. In addition, the present study emphasized exploration of the school-age children’s health beliefs. Further study is required to examine the interplay between school-age children’s health beliefs and the socioeconomic circumstances of their families.

In conclusion, children held misconceptions regarding behavioral, environmental and genetic risk factors. They also perceived obstacles that threaten their ability to engage in preventive behaviors. Health education interventions may enhance school-age children’s health literacy and be translated into preventive action. Overcoming practical challenges such as shortage of footwear and other hygiene facilities requires other forms of interventions such as livelihood-strengthening activities. Linking podoconiosis-affected families with local governmental or non-governmental organizations providing socio-economic support for households may assist school-age children in those families to sustainably engage in preventive behaviors.
